# The improvement of QRS-T angle as a manifestation of reverse electrical remodeling following renal transplantation in end-stage kidney disease patients on haemodialysis

**DOI:** 10.1186/s12882-019-1624-3

**Published:** 2019-12-02

**Authors:** Andrzej Jaroszyński, Jacek Furmaga, Tomasz Zapolski, Tomasz Zaborowski, Sławomir Rudzki, Wojciech Dąbrowski

**Affiliations:** 1 Institute of Medical Sciences, Jan Kochanowski University in Kielce, Al. IX Wieków Kielc 19A, 25-317 Kielce, Poland; 2 Department of Family Medicine, Medical University of Lublin, Lublin, Poland; 3 Department of General and Transplant Surgery and Nutritional Treatment, Medical University of Lublin, Lublin, Poland; 4 Department of Cardiology, Medical University of Lublin, Lublin, Poland; 5 Department of Anesthesiology and Intensive Care, Medical University of Lublin, Lublin, Poland

**Keywords:** QRS-T angle, Renal transplantation, Hypervolemia, Ejection fraction, Reverse remodeling

## Abstract

**Background:**

Successful renal transplantation (RT) reverses some of the cardiac changes and reduces cardiac mortality in hemodialysis (HD) patients. Widened QRS-T angle reflects both ventricular repolarization and depolarization. It is considered a sensitive and strong predictor of heart ventricular remodeling as well as a powerful and independent risk stratifier suitable in predicting cardiac events in various clinical settings. The study aimed to assess the influence of the RT on QRS-T angle and to evaluate factors influencing QRS-T changes in renal transplanted recipients (RTRs).

**Methods:**

Fifty-four selected HD patients who have undergone RT were included. Blood chemistry, echocardiography, and QRS-T angle were evaluated 5 times: about 1 week, 3 months, 6 months, 1 year and 3 years after RT.

**Results:**

An improvement of echocardiographic parameters was observed. The dynamics of changes in individual parameters were, however, variable. QRS-T angle correlated with echocardiographic parameters. The biphasic pattern of the decreases of QRS-T angle was observed. The first decrease took place in the third month of follow-up. The second decrease of QRS-T angle was observed after 1 year of follow-up. The QRS-T angle was higher in RTRs compared with controls during each evaluation. Multivariable analysis demonstrated that the decrease of left ventricle enddiastolic volume was an independent predictor of early QRS-T angle improvement. The increase of left ventricle ejection fraction was found to be the independent predictor of the late QRS-T angle improvement.

**Conclusions:**

RT induces biphasic reverse electrical remodeling as assessed by the narrowing of QRS-T angle. Early decrease of QRS-T angle is mainly due to the normalization of volume status, whereas late decrease is associated predominantly with the improvement of cardiac contractile function.

## Background

The pathology of cardiovascular (CV) disease is complex and multifactorial in patients with end-stage renal disease (ESRD) patients. Both traditional and nontraditional risk factors induce structural as well as functional changes of the myocardium, contributing to myocardial remodeling and cardiomyopathy, resulting in substantially increased cardiac events and death. Successful renal transplantation (RT) reverses some of the cardiac changes observed in end-stage renal disease (ESRD) patients, and reduces CV mortality. CV death, including sudden cardiac death (SCD), however, is still much higher compared with the general population [1–7].

**Fig. 1:**
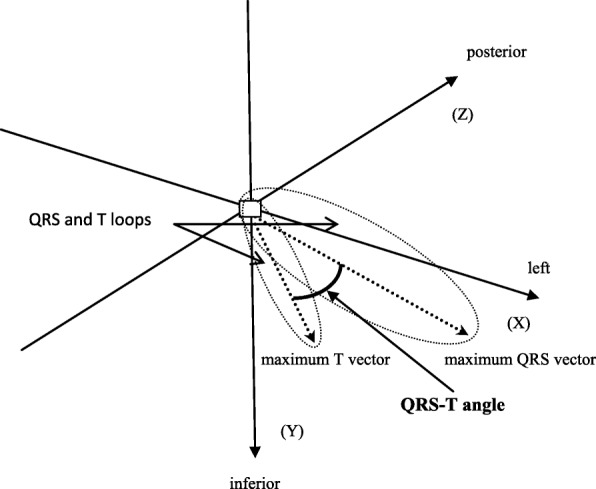
QRS-T angle – the spatial angle between the vectors of the T-wave and QRS loops

The QRS-T angle is the spatial angle between the vectors of the T-wave and QRS loops (Fig. 1). A widened QRS-T angle is considered a cumulative measurement of cardiac electrical activity, reflecting ventricular repolarization as well as ventricular depolarization. Depolarization abnormalities reflect pathology of ventricular structures, while repolarization abnormalities reflect myocardial action potential homogeneity abnormalities, resulting in electrical instability [8–14]. A widened QRS-T angle is considered a sensitive and strong predictor of heart ventricular structural as well as electrical remodeling [15]. Many studies have confirmed that QRS-T angle is an independent and strong predictor of adverse cardiac events both in the general population [10, 13] and other patient groups [11, 14, 16–18], including dialysis patients [8, 19–22]. Moreover it is especially useful for the prediction of SCD [9–17].

To the best of our knowledge there are no data in the literature on the influence of RT on QRS-T angle. The objective of the study was to: (i) assess the influence of the RT on QRS-T angle and (ii) evaluate the possible factors influencing QRS-T changes in a group of selected renal transplanted recipients (RTRs).

## Methods

### Patients

The study included adult patients transplanted in Lublin center (Poland). The exclusion criteria were: manifest coronary artery disease, implanted pacemaker or implantable cardioverter defibrillator, atrial fibrillation, and unstable graft function during the follow-up. Unstable graft function was defined as delayed graft function of more then 3 days, acute graft rejection, and acute kidney injury defined by the RIFLE criteria.

All patients gave written consent, and the studies were conducted in accordance with the Declaration of Helsinki, and approved by members of the Bioethics Committee of Medical University in Lublin, Poland (KE-254/125/2011).

Patients were followed for 3 years after the day of RT. All patients were evaluated 5 times: about 1 week, 3 months, 6 months, 1 year and 3 years after RT. During the subsequent phases the following measurements were performed:

### Echocardiographic examination

Transthoracic echocardiographic examination was performed (according to the recommendations of the American Society of Echocardiography) by an cardiologist, who was blinded to the clinical data of the study subjects [23, 24]. On the basis of the planimetric measurements, the following parameters were calculated: left ventricle endsystolic and enddialstolic volumes (LVESV and LVEDV, respectively), left ventricle stroke volume (SV), stroke index (SI), cardiac output (CO), cardiac index (CI), left ventricle ejection fraction (EF), left ventricle mass (LVM), and left ventricle mass index (LVMI), left atrial volume (LAV), LA volume index (LAVI). The body surface area was calculated according to the Gehan and George formula [25].

### ECG, derived vectorcardiogram (VCG)

Surface 12-lead resting ECG was recorded using a Cardiax device (IMED Co Ltd., Budapest, Hungary). The recordings were automatically transformed into three orthogonal leads X, Y and Z according to the inverse Dower method. Next, the value for the QRS/T angle was automatically calculated from the maximum spatial QRS and T vectors by using Cardiax commercial software. An abnormal spatial QRS-T angle was defined as a spatial QRS-T angle > 116 for females and a spatial QRS-T angle > 130 in male subjects [26]. The QRS-T angle was also evaluated in 60 healthy controls (hospital employees) who came for routine physical check-ups. The controls did not show any abnormalities in the physical examination, ECG, and laboratory test. The group’s gender distribution and age range were similar to the group of patients. In the control group, the QRS-T angle value was evaluated at the same intervals as in patients.

### Statistical analysis

The Kolmogorov-Smirnov test was used to test the normality of the distribution of the results. Normally distributed variables were expressed as mean ± SD, while non-normally distributed variables were expressed as median and range. Continuous data were compared using the Student t-test for paired data when normally distributed and using Mann-Whitney U-test when non-normally distributed. In order to compare the results between more than two groups, one-way ANOVA was used. Linear regression analysis was performed by using the Pearson test. Multivariable logistic regression analysis was performed to identify the independent determinants of QRS-T changes induced by the RT process. Explanatory variables with a *p* value ≤0.15 in the univariate analysis were entered into a multivariate analysis. A *p*-value < 0.05 was considered significant.

## Results

Of the total of 92 available RTRs patients that were transplanted in the last 4 years in our center, 38 patients were excluded due to unstable graft function, manifest coronary artery disease, lack of all echocardiographic measurements, implanted pacemaker or implantable cardioverter defibrillator as well as less than 3 years follow-up. The remaining 54 patients, aged 44.6 ± 8.9 years, including 28 women aged 46.7 ± 9.4 years and 26 men aged 42.6 ± 8.7 years were followed-up for 3 years. The causes of ESRD were: glomerulonephritis (*n* = 20), diabetes (*n* = 9), obstructive nephropathy (*n* = 6), tabulo-interstitial nephritis (*n* = 4), vasculitis (*n* = 2), polycystic kidney disease (*n* = 3) and unknown/uncertain (*n* = 10). All patients have undergone RT from unrelated deceased donors. The immunosuppresion regimen included methylprednisolone (100%), mycophenolate mofetil (96,3%) and cyclosporine (35.2%) or tacrolimus (64.8%). Table 1 lists the baseline characteristics of the study population.

**Table 1:** Baseline characteristics (*n* = 54)

Characteristic	Value
age [years]	44.6 ± 8.9
gender (men/women) [*n* (%)]	26 (48.1)/28 (51.9)
time on dialysis until RT [months]	36.65 (±19.34)
haemoglobin [g/dL]	12.43 (±2.89)
sodium [mmol/L]	138.4 (±2.01)
potassium [mmol/L]	4.74 (±1.20)
magnesium [mmol/L]	0.98 (±0.25)
calcium [mmol/L]	2.49 (±0.26)
phosphorus [mmol/L]	1.63 (±0.47)
parathormone [pg/mL]	695.3 (±666.8)
creatinine [μmol/L]	567.2 (±229.3)
urea [mmol/L]	9.54 (±5.34)
total protein [g/L]	74.8 (±13.8)
albumin [g/L]	4.5 (±1.11)
hs-CRP [mg/L]	3.42 (±2.02)
total cholesterol [mg/dL]	223.0 (±62.2)
LDL-cholesterol [mg/dL]	138.0 (±42.18)
HDL-cholesterol [mg/dL]	68.0 (±22.05)
triglycerides [mg/dL]	211.2 (±65.69)
glucose [mg/dL]	92.56 (±42.18)
troponin T [μg/L]	0.019 (±0.035)
treatment prior to RT	
ACE-inhibitors/sartans [*n* (%)]	36 (66.7)
calcium blockers [*n* (%)]	42 (77.8)
beta-blockers [*n* (%)]	41 (75.9)
alfa-blockers [*n* (%)]	4 (7.4)
clonidine [*n* (%)]	2 (3.7)
statins [*n* (%)]	26 (48.1)
immunosuppression	
methylprednisolone [*n* (%)]	54 (100)
mycophenolate mofetil [*n* (%)]	52 (96.3)
cyclosporine [*n* (%)]	18 (33.3)
tacrolimus [*n* (%)]	36 (66.6)

hs-CRP – high-sensitivity C-reactive protein; ACE- inhibitors –angiotensin converting enzyme inhibitors; sartans - angiotensin 2 receptor blockers

An analysis of changes in heart rate (HR) and blood pressures are depicted in Table 2. During the follow-up regular decrease of HR was observed and at the end of the observation HR was lower than at the baseline. During the follow-up systolic blood pressure (SBP) steadily decreased, while diastolic blood pressure (DBP) did not change. As a result, the pulse pressure (PP) decrease was noted.

**Table 2:** Heart rate and blood pressures in patients after RT (*n* = 54)

parameter	1 week after RT (1)	3 months after RT (2)	6 months after RT (3)	1 year after RT (4)	3 years after RT (5)	*p*						
						1vs2	1vs3	1vs4	1vs5	2vs3	3vs5	4vs5
HR [n/min]	77.2 ± 8.9	75.1 ± 7.7	75.3 ± 7.9	73.2 ± 8.0	71.6 ± 7.3	0.378	0.381	0.098	0.014	0.872	0.062	0.373
SBP [mmHg]	138.2 ± 12	135.3 ± 12	135.8 ± 11	134.2 ± 14	132.9 ± 10	0.019	0.021	0.005	< 0.001	0.692	0.109	0.112
DBP [mmHg]	87.2 ± 8.0	86.9 ± 9.3	86.5 ± 8.8	86.9 ± 8.7	87.1 ± 7.5	0.691	0.543	0.552	0.569	0.782	0.577	0.853
PP [mmHg]	51.1 ± 8.9	51.6 ± 8.2	49.3 ± 9.2	47.2 ± 8.1	45.7 ± 5.3	0.864	0.231	0.002	< 0.001	0.267	0.012	0.203

*HR* Heart rate, *SBP* Systolic blood pressure, *DBP* Diastolic blood pressure, *PP* Pulse pressure

The analysis of drug treatment is shown in Table 3. During observation, a reduction in the use of calcium channel blockers was observed. We did not observe differences in the use of both beta-blockers and angiotensin-converting enzyme inhibitors/aldosterone receptors blockers.

**Table 3:** Pharmacological treatment in patients after RT (*n* = 54)

parameter	1 week after RT (1)	3 months after RT (2)	6 months after RT (3)	1 year after RT (4)	3 years after RT (5)	*p*						
						1vs2	1vs3	1vs4	1vs5	2vs3	3vs5	4vs5
ACEI/ARB [*n*(%)]	37 (68.5)	38 (70.1)	38 (70.1)	37 (68.5)	39 (72.2)	0.791	0.731	1.000	0.572	1.000	0.795	0.6576
CCB [*n*(%)]	40 (74.1)	36 (66.6)	33 (61.1)	35 (64.8)	32 (59.2)	0.104	0.017	0.009	< 0.001	0.108	0.423	0.073
BB [*n*(%)]	41 (75.9)	39 (72.2)	39 (72.2)	39 (72.2)	40 (74.1)	0.512	0.512	0.512	0.517	1.000	0.789	0.790

*ACEI* Angiotensin-converting enzyme inhibitors, *ARB* Aldosterone receptors blockers, *BB* Beta-blockers

Gradually reduction of planimetric parameters of the LV was observed. At the end of the observation, all evaluated parameters were lower in comparison with the baseline, however, the dynamics of changes in individual parameters varied depending on the parameters evaluated in subsequent observation periods (Table 4). Reduction of planimetric dimensions was associated by the change of LVH parameters, however, these changes started to be significant only after 1 year of observation (Table 4). Due to the planimetric indices reduction, the decrease in LV volumetric parameters was noted. The dynamics of changes in individual parameters were, however, variable (Table 4). Contractility parameters of LV also improved, but the significance of these changes started after 1 years of follow-up (Table 4).

**Table 4:** Echocardiographic parameters in patients after RT (*n* = 54)

parameter	1 week after RT (1)	3 months after RT (2)	6 months after RT (3)	1 year after RT (4)	3 years after RT (5)	*p*						
						1vs2	1vs3	1vs4	1vs5	2vs3	3vs5	4vs5
LV dimensions												
LVEDd [mm]	52.3 ± 5.0	49.8 ± 4.4	50.0 ± 4.7	49.9 ± 4.6	49.5 ± 4.7	0.012	0.016	0.001	< 0.001	0.953	0.079	0.232
LVESd [mm]	35.2 ± 5.4	34.1 ± 3.6	34.3 ± 4.2	33.8 ± 4.8	32.2 ± 4.6	0.093	0.108	0.009	0.002	0.580	0.015	0.016
PWDd [mm]	12.2 ± 1.4	12.0 ± 1.4	11.7 ± 1.3	11.7 ± 1.7	11.0 ± 1.3	0.872	0.009	0.016	< 0.001	0.684	0.011	0.072
PWSd [mm]	15.4 ± 1.1	16.2 ± 1.6	16.4 ± 2.0	16.1 ± 2.1	17.2 ± 2.1	0.129	0.098	0.353	0.001	0.657	0.011	0.016
IVSDd [mm]	13.5 ± 1.3	13.2 ± 0.8	13.1 ± 0.8	12.0 ± 0.7	11.7 ± 1.2	0.362	0.245	0.002	< 0.001	0.421	0.017	0.048
IVSSd [mm]	16.2 ± 1.4	16.4 ± 1.2	17.8 ± 2.2	16.9 ± 2.1	17.5 ± 1.8	0.675	0.002	0.021	< 0.001	0.173	0.534	0.426
LVM indices												
LVM [g]	321 ± 70	323 ± 74	320 ± 71	309 ± 73	281 ± 65	0.866	0.354	0.011	< 0.001	0.191	< 0.001	0.003
LVMI [g/m2]	171 ± 36	171 ± 33	170 ± 34	162 ± 33	1459 ± 31	0.884	0.443	0.009	< 0.001	0.124	< 0.001	0.002
LV volumetric parameters												
LVEDV [ml]	135 ± 28	125 ± 30	122 ± 29	121 ± 34	117 ± 29	0.004	0.005	0.002	0.001	0.764	0.043	0.121
LVESV [m]	53.9 ± 19.9	50.1 ± 13.5	50.4 ± 15.6	48.5 ± 18.5	42.3 ± 15.3	0.059	0.118	0.021	0.005	0.439	0.008	0.011
SV [ml]	81.6 ± 17.1	78.2 ± 14.4	77.6 ± 17.1	76.4 ± 21.0	76.1 ± 18.3	0.009	0.011	0.014	0.008	0.489	0.706	0.601
Cardiac output parameters												
CO [l/min]	6.09 ± 0.73	5.80 ± 0.69	5.72 ± 0.70	5.51 ± 0.78	5.51 ± 0.63	0.031	0.010	0.008	0.003	0.664	0.212	0.923
CI [l/min/m2]	3.31 ± 0.49	3.11 ± 0.55	3.09 ± 0.47	2.96 ± 0.46	2.96 ± 0.52	0.019	0.014	0.011	0.006	0.821	0.263	0.976
Contractility parameters												
EF [%]	60.9 ± 9.9	59.1 ± 8.8	60.8 ± 9.1	65.3 ± 8.9	65.5 ± 8.8	0.892	0.859	0.002	0.001	0.233	0.002	0.601

*LVEDd* Left ventricle enddiastolic diameter, *LVESd* Left ventricle endsystolic diameter, *PWSd* Posterior wall systolic diameter; *PWDd* Posterior wall diastolic diameter, *IVSSd* Interventricular septum systolic diameter, *IVSDd* Interventricular septum diastolic diameter, *LVESV* Left ventricle endsystolic volume, *LVEDV* Left ventricle enddiastolic volume, *SV* Left ventricle stroke volume, *SI* Stroke index, *CO* Cardiac output, *CI* Cardiac index, *EF* Left ventricle ejection fraction, *LVM* Left ventricle mass, *LVMI* Left ventricle mass index

A detailed analysis of the changes in the LA planimetric as well as volumetric indices are shown in Table 3. During the follow-up planimetric parameters showed a constant and progressive reduction in dimensions. The volumetric indices followed the described changes confirming a significant reduction in the LA dimensions (Table 4).

**Fig. 2:**
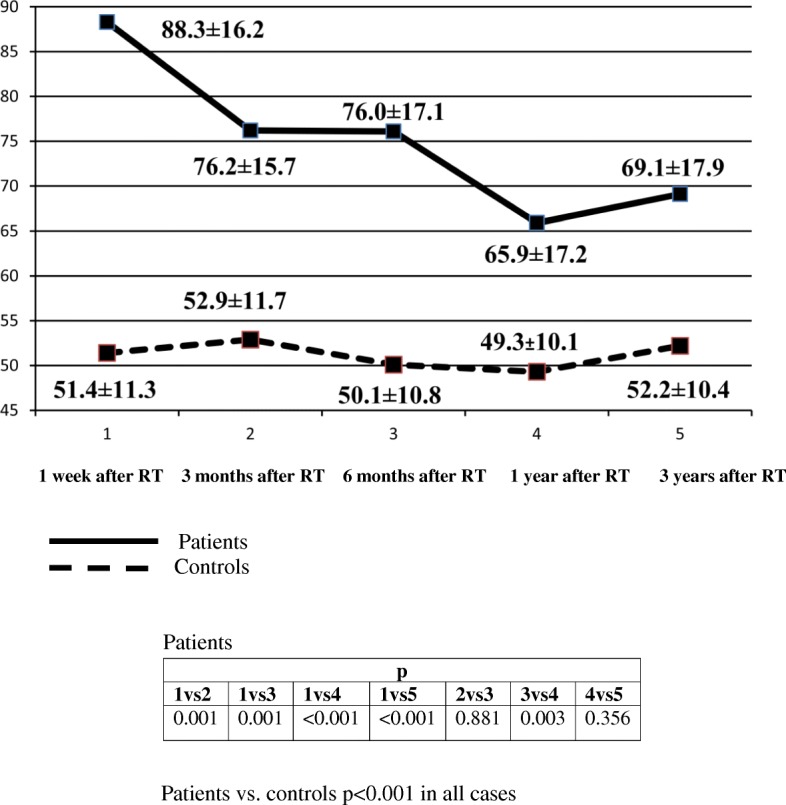
QRS-T angle in patients after RT and in the control group

A detailed analysis of changes in QRS-T angle are depicted in Fig. 2. At baseline abnormal QRS-T angle was found in 10 (18.5%) patients. In 32 (59.3%) patients QRS-T angle was > 50° and lower than abnormal value, whereas in 12 (22.2%) patients it was < 50°. The biphasic pattern of the decreases of QRS-T angle was observed. The first decrease took place in the 3 month of follow-up. Next, QRS-T angle remained stable to 1 year after RT. The second decrease of QRS-T angle was observed after 1 year of follow-up. Abnormal QRS-T angle was then found in 7 (13.0%) patients (all patients had abnormal QRS-T angle at baseline). In 33 (61.1%) patients QRS-T angle was > 50 and lower than abnormal value, whereas in 14 (25.9%) patients it was < 50°. In the final phase of follow-up an unsignificant increase of QRS-T angle was found. The QRS-T angle was lower in controls (51.4 ± 11.3) compared with RTRs at the beginning of the observation and throughout the entire observation (*p* < 0.001 in all cases). In the control group, no significant changes in QRS-T angle values were observed during observation (Fig. 2).

Correlations between QRS-T angle and some echocardiographic indices (both 1 week after RT and 1 year after RT) are depicted in Table 5. Both 1 week after RT and 1 year after RT significant correlations were found between QRS-T angle and LVEDV, LVMI, SV, CI, and EF.

**Table 5:** Correlations of QRS-T angle with LV structural indices 1 week as well as 1 year after RT

	QRS-T angle			
	1 week after RT		1 year after RT	
	*r*	*p*	*r*	*p*
LVEDV	0.53	< 0.001	0.51	< 0.001
LVMI	0.39	0.008	0.37	0.011
SV	- 0.33	0.026	- 0.31	0.029
EF	- 0.57	< 0.001	- 0.59	< 0.001

*LVEDV* Left ventricle enddiastolic volume, *LVMI* Left ventricle mass index, *SV* Left ventricle stroke volume, *EF* Left ventricle ejection fraction

Multivariable logistic regression analysis was performed to indentify determinants of QRS-T angle changes observed 3 months as well as 1 year after RT. The results are shown in Table 6. Multivariable analysis demonstrated that the decrease of LVEDV was the independent predictor of QRS-T angle improvement 3 months after the RT. One year after the RT the increase of LVEF was found to be the independent predictor of QRS-T angle improvement.

**Table 6:** Multivariable logistic regression analysis in order to indentify determinants of QRS-T angle changes induced after (A) 3 months as well as after (B) 1 year of RT

Parameter	Univariate	*p*	Multivariate	*p*
A				
Age	1.23 (0.89–1.1.81)	0.013	1.31 (1.05–2.62)	0.101
Beta-blockers	1.29 (0.78–2.87)	0.211		
Δ Systolic blood pressure	1.14 (0.72–1.65)	0.143	1.18 (0.69–2.35)	0.237
Baseline troponin T	2.89 (2.03–3.65)	0.126	2.43 (1.78–3.97)	0.198
Δ LVEDd	2.35 (1.09–3.26)	0.201		
Δ LVEDV	1.38 (1.11–1.67)	0.004	1.29 (0.88–1.49)	0.006
Δ LVESV	2.09 (0.93–3.33)	0.241		
Δ SV	0.99 (0.66–1.22)	0.011	1.12 (0.69–1.59)	0.126
Δ CO	1.47 (0.75–2.36)	0.231		
B				
Diabetes mellitus	1.37 (0.88–2.06)	0.137	1.25 (0.74–2.99)	0.314
Δ LVM	1.71 (1.07–2.34)	0.228		
Δ LVEDd	1.09 (0.78–2.17)	0.233		
Δ LVEF	2.67 (1.89–2.45)	0.006	2.11 (1.72–2.63)	0.002
Δ LAVI	2.34 (1.86–2.82)	0.023	1.98 (1.54–2.33)	0.213

*LVEDd* Left ventricle enddiastolic diameter, *LVESV* Left ventricle endsystolic volume, *LVEDV* Left ventricle enddiastolic volume, *SV* Left ventricle stroke volume, *CO* Cardiac output, *CI* Cardiac index, *LVM* Left ventricular mass, *LVEF* Left ventricular ejection fraction, *LAVI* left atrium volume index. Exclusively parameters with a *p* value < 0.15 at the univariate analysis are shown in order to improve readability

## Discussion

Three key findings were generated in our study: (i) RT improves QRS-T angle in a biphasic manner, however, QRS-T values are still higher than in controls, (ii) the first decrease takes place in the short term after RT, and is associated with the improvement of volume dependent parameters, (iii) the second QRS-T angle decrease occurs 1 year after RT, and is predominantly associated with the improvement of cardiac contractile function.

QRS-T angle is a strong, independent and reliable predictor of CV mortality in various populations, including HD patients. It provides greater prognostic value than any of the commonly utilized ECG indicators [8, 9, 17, 27], however, that there is no consensus on the methods for the calculation of the QRS-T angle and the cut-point value that defines abnormal QRS-T angle depends on the method of QRS-T angle estimation. It may cause difficulties in comparing results of different studies. To our knowledge, ours is the first study that shows that RT improves QRS-T angle, however, its values are still higher than in controls, suggesting persistent higher CV risk. It is in agreement with previous studies showing that RT reduce cardiac mortality, however, it is still higher in RTRs compared to the general population [2–4].

Given the high CV risk of RTR patients, it is of clinical importance to identify not only patients at high risk but also to identify determinants of an abnormal QRS-T angle that potentially might represent therapeutic targets to improve prognosis in this patients’ population.

Our study has revealed that the short term QRS-T angle decrease is associated with the improvement of LVEDV. In HD patients it is well established that chronic fluid overload leads to increased CV mortality due to hypertension, left ventricular dysfunction, heart failure, and arrhythmias, including SCD [28–30]. Widespread consensus exists that LVEDV is a volumetric parameter of the left ventricle and reflects preload. Greater LVEDV values cause greater distention of the ventricle. Myocardial stretch due to volume overload leads to electrophysiological abnormalities in refractoriness and conduction, essential components of both re-entry and proarrhythmia, and can provoke arrhythmias [31, 32]. Increased LVEDV reflecting the presence of a dilated ventricle has been associated with increased risk of CV death both in HD patients and in RTRs [28, 33]. In HD patients hypervolemia is universal, however, both the prevalence and consequences of fluid overload in RTRs have not been intensively investigated. Chan et al. [31] using multifrequency bioimpedance analysis have found that hypervolemia is common among clinically stable RTRs, and is observed in 30% of them, with 5% classified as severe hypervolemia. Given that hydration status is a potentially modifiable factor the relation between QRS-T angle and LVEDV is of interest. Its clinical significance, however, needs further prospective follow-up investigations.

In the present study we have observed the improvement of LV structural parameters, suggesting myocardial reverse remodeling process due to RT. We have also demonstrated that late QRS-T angle decrease is predominantly associated with the improvement of cardiac contractile function. The restoration of the renal function following RT, induces myocardial reverse remodelling, the reparative processes leading to partial restitution of the myocardial structure and function [5, 34]. However, LV structural indices correlated with QRS-T angle only moderately. This may suggest that LV structural improvement is only partly responsible for the reverse of inhomogeneities of the myocardium repolarisation phase in RTRs. This implies that other mechanisms should be considered as responsible for electrical remodeling, independently from structural changes. This issue needs further investigation.

Most, however, not all studies [35] show that RT improves EF, functional status of heart failure as well as increases RTRs survival [1, 5, 34, 36, 37]. In our study we found slight, however significant improvement of LVEF after 1 year after RT. Growing evidence suggests that the greatest benefit of RT in relation to the LVEF refers to RTRs with advanced systolic heart failure [23, 34]. In the present study LVEF was in normal range in most RTRs, thus the increase of LVEF was slight only. The relation between LVEF and QRS-T angle have been commonly observed both in the general population and in HD patients [8, 9, 11]. Recently published study using MRI showed that QRS-T angle is associated with left ventricle function, mass as well as myocardial scar burden [15]. It has also been suggested that widened spatial QRS-T angle may be a sensitive and strong predictor of heart ventricular electrical remodelling, which may display early, before ECG or echocardiographic signs of left heart damage become evident [15]. The relationship found in our study between the slight increase of EF and the improvement of QRS-T angle values suggests that even slight improvement of left ventricular function remains of importance and might represent a potential therapeutic target.

The present study has some limitations. First, in this study we did not assess outcome data. Given the previously established relationship between the spatial QRS-T angle and cardiac events, and especially SCD we can only present a hypothesis that the QRS-T angle improvement may reflect the reverse of inhomogeneities of the myocardium repolarisation phase in RTRs. Further studies using clinical endpoints would allow for more definitive conclusions. Second, present study is a clinical cohort study, thus election bias may have played a role in influencing the results. Third, the numbers of patients was relatively small, nevertheless was large enough to demonstrate that RT improves QRS-T angle.

## Conclusions

RT induce biphasic reverse electrical remodeling as assessed by the narrowing of QRS-T angle. Early decrease of QRS-T angle is mainly due to the normalization of volume status, whereas late decrease is associated predominantly with the improvement of cardiac contractile function.

## Data Availability

All data used in the current study are presented in the manuscript or available upon request. To request the data, please contact the corresponding author.

## Abbreviations

CI: Cardiac index
CO: Cardiac output
CV: Cardiovascular
DBP: Diastolic blood pressure
EF: Left ventricle ejection fraction
ESRD: End-stage renal disease
HD: Hemodialysis
HR: Heart rate
LAV: Left atrial volume
LAVI: Left atrial volume index
LVEDV: Left ventricle and enddialstolic volume
LVESV: Left ventricle endsystolic volume
LVM: Left ventricle mass
LVMI: Left ventricle mass index
PP: Pulse pressure
RT: Renal transplantation
RTRs: Renal transplanted recipients
SBP: Systolic blood pressure
SCD: Sudden cardiac death
SI: Stroke index
SV: Left ventricle stroke
